# A Novel Distal Enhancer Mediates Inflammation‐, PTH‐, and Early Onset Murine Kidney Disease‐Induced Expression of the Mouse *Fgf23* Gene

**DOI:** 10.1002/jbm4.10023

**Published:** 2017-11-21

**Authors:** Melda Onal, Alex H Carlson, Jeff D Thostenson, Nancy A Benkusky, Mark B Meyer, Seong M Lee, J Wesley Pike

**Affiliations:** ^1^ Department of Biochemistry University of Wisconsin‐Madison Madison WI USA; ^2^ Department of Physiology and Biophysics University of Arkansas for Medical Sciences Little Rock, AR USA; ^3^ Department of Biostatistics University of Arkansas for Medical Sciences Little Rock AR USA

**Keywords:** PTH/VIT D/FGF23, CYTOKINES, OSTEOCYTES, TRANSCRIPTIONAL REGULATION, DISORDERS OF CALCIUM/PHOSPHATE METABOLISM, GENETIC ANIMAL MODELS

## Abstract

Fibroblast growth factor 23 (FGF23) production is regulated by both calciotropic hormones and inflammation. Consistent with this, elevated FGF23 levels are associated with inflammatory markers as well as parathyroid hormone (PTH) in various disease states, including chronic kidney disease (CKD). However, the molecular mechanisms underpinning *Fgf23* transcription in response to these regulators are largely unknown. We therefore utilized chromatin immunoprecipitation followed by DNA sequencing (ChIP‐seq) data from an osteocyte cell line to identify potential regulatory regions of the *Fgf23* gene. Based on ChIP‐seq analysis of enhancer‐associated histone modifications, including H3K4 methylation and H3K9 acetylation, we discovered several potential enhancers for *Fgf23*, one of which was located 16kb upstream of the gene's transcriptional start site. Deletion of this putative enhancer from the mouse genome using CRISPR‐Cas9 technology led to lower bone, thymus, and spleen expression of *Fgf23* mRNA without altering circulating levels of the intact hormone, although as previously reported, only bone displayed significant basal expression. Nevertheless, lack of the −16kb enhancer blunted FGF23 upregulation in a tissue‐specific manner by the acute inflammatory inducers lipopolysaccharide (LPS), interleukin‐1‐beta (IL‐1β), and tumor necrosis factor‐alpha (TNFα) in bone, non‐osseous tissues, and in circulation. Lack of the −16kb enhancer also inhibited PTH‐induced bone *Fgf23* mRNA. Moreover, the absence of this *Fgf23* enhancer in an oxalate diet‐induced murine CKD model prevented the early onset induction of osseous, renal, and thymic *Fgf23* mRNA levels and led to a significant blunting of elevated circulating intact FGF23 levels. These results suggest that −16kb enhancer mediates the induction of *Fgf23* by inflammation and PTH and facilitates the increase in FGF23 expression in a murine model of CKD. As exemplified herein, these *Fgf23* enhancer‐deleted mice will provide a unique model in which to study the role of FGF23 expression in inflammatory diseases. © 2017 The Authors. *JBMR Plus* is published by Wiley Periodicals, Inc. on behalf of the American Society for Bone and Mineral Research.

## Introduction

Fibroblast growth factor 23 (FGF23) is a bone‐derived hormone that regulates phosphate homeostasis and vitamin D metabolism. FGF23 protein is synthesized as a full‐length, biologically active 32kDa glycoprotein that is biologically inactivated through cleavage by furin and furin‐like convertases.^1^, ^2^, ^3^, ^4^ Under physiological conditions, increased serum phosphate levels function to upregulate FGF23, which acts in turn at the kidney through both fibroblast growth factor receptors (FGFRs) and the co‐receptor alpha Klotho (αKlotho) to decrease levels and activity of the sodium phosphate cotransporters NPT2a and NPT2c.^5^ This action inhibits renal phosphate reabsorption^6^ and alters the metabolism of 1,25‐dihydroxyvitamin D_3_ (1,25(OH)_2_D_3_).^2^

Defects in the production or cleavage of FGF23 lead to altered phosphate and vitamin D levels in various diseases such as familial tumoral calcinosis (TC)^7^ or autosomal‐dominant hypophosphatemic rickets (ADHR).^3^ Elevated FGF23 levels have also been detected in various other diseases in which the role or the mechanism of its elevation have not been firmly established, including chronic kidney disease (CKD)^8^, ^9^, ^10^ and inflammatory maladies such as inflammatory bowel disease.^11^ For example, FGF23 levels increase in early stages of renal failure and become gradually elevated during CKD progression in parallel with increased serum phosphate levels.^12^ Elevated FGF23 levels in CKD have also been associated with increased cardiovascular disease and mortality.^8^, ^13^, ^14^ Although these studies suggest that FGF23 is an important component in CKD, the causes, sources, and the molecular mechanisms through which FGF23 is increased are largely unknown.

Injections of pro‐inflammatory cytokines and sepsis have also been shown to be linked to hypophosphatemia,^15^, ^16^, ^17^ suggesting a potential interplay between phosphate, inflammation, and FGF23. Consistent with these findings, studies by Atkins and colleagues have shown that inflammation increases transcription of both mouse *Fgf23* and human *FGF23* genes in both the murine osteocytic cell line (IDG‐SW3) and in human bone chips, respectively.^18^ Moreover, in vivo studies by Wolf and colleagues have shown that both acute and chronic inflammation induced by single or repeated injections of bacteria or interleukin‐1‐beta (IL‐1β) increase bone *Fgf23* mRNA production.^19^ Because osteocytes produce the highest levels of basal *Fgf23*, both of these studies have focused on osseous sources of FGF23.^1^ Despite this, *Fgf23* is also expressed at lower levels in other tissues and cell types, including the thymus, spleen, brain, activated macrophages, and dendritic cells.^1^, ^20^, ^21^, ^22^ Masuda and colleagues has shown that inflammatory stimuli can increase *Fgf23* expression of activated dendritic cells and macrophages.^21^ Consistent with this, Fanti and colleagues recently reported that upon acute or chronic exposure to low doses of lipopolysaccharides (LPS), spleen FGF23 production increased significantly.^23^ Although these studies establish that inflammation increases osseous and spleen‐derived FGF23 production, the contribution of non‐osseous tissues to FGF23 production and its pathophysiological role in inflammatory diseases are unknown.

FGF23 is expressed in many cell types^21^, ^22^, ^24^, ^25^ and is regulated by hormones such as 1,25(OH)_2_D_3_ 26, 27, 28 and cytokines such as IL‐1β.^18^ Studies to date that have explored the underlying mechanisms of this regulation have utilized a combination of (i) transient transfection techniques that are limited to an evaluation of the activity of promoter‐proximal regions attached to a reporter and/or (ii) the use of selected pathway inhibitors. These methods have revealed roles for nuclear factor kappa β (NF‐κB)^18^, ^21^ and hypoxia‐inducible factor 1α (HIF1α)^19^, ^29^ binding at the *Fgf23* promoter region, suggesting that they might mediate inflammation‐induction of *Fgf23* transcription. In both cases, however, inhibitors of these transcriptional activators failed to prevent the induction in *Fgf23* mRNA levels by inflammation and do not provide any in vivo evidence for the gene's direct regulation, highlighting the need for unbiased identification of *Fgf23* transcriptional regulatory regions.

Advances in genome‐wide techniques such as chromatin immunoprecipitation followed by DNA sequencing (ChIP‐seq) have revealed that the transcriptional regulation of many genes, including receptor activator of NF‐κB ligand (*Tnfsf11*), vitamin D receptor (*Vdr*), or matrix metallopeptidase 13 (*Mmp13*), are mediated through a complex network of enhancers each providing distinct factor‐ and cell‐type specificity.^30^, ^31^, ^32^, ^33^, ^34^, ^35^, ^36^ More importantly, these studies have shown that although promoter‐proximal regions may play a role in regulation, many if not most regulatory components (enhancers) reside distal to the promoter.^31^, ^33^, ^34^, ^35^, ^36^ To determine the locations of regulatory regions of the *Fgf23* gene, we utilized prior genome‐wide ChIP‐seq analyses of CTCF occupied sites, histone enrichment marks associated with chromatin structure, and enhancers to identify potential enhancers of *Fgf23*. We deleted one of the identified putative enhancers, which was located in an intergenic region 16kb upstream of *Fgf23*, from the mouse genome using clustered regularly interspaced short palindromic repeats (CRISPR)‐CRISPR‐associated protein‐9 nuclease (Cas9) (CRISPR‐Cas9) technology and explored the consequence of this deletion in the newly developed mouse strain. Lack of this putative enhancer decreased basal *Fgf23* mRNA levels in bone, spleen, and thymus, but did not alter the levels of circulating intact FGF23 (iFGF23) nor did it affect the expression of FGF23 target genes in kidney, bone, or distinct parameters of mineral homeostasis. However, mice lacking the −16kb enhancer showed a significantly blunted FGF23 response to LPS and the inflammatory cytokines IL‐1β and tumor necrosis factor‐alpha (TNFα) with evident tissue specificity. Deletion of the −16kb enhancer also prevented the early onset induction of renal, thymic, and osseous *Fgf23* mRNA levels and blunted the increase in circulating iFGF23 in an oxalate‐induced model of CKD. Because both inflammation and parathyroid hormone (PTH) are potential causes of increased FGF23 levels in early stages of CKD, we also show that PTH mediates bone *Fgf23* transcription via the −16kb enhancer. These results suggest that a novel distal *Fgf23* enhancer located −16kb upstream of the gene's transcriptional start site (TSS) mediates inflammatory and PTH induction of FGF23 and is required for FGF23 upregulation in early phases of CKD.

## Materials and Methods

### Reagents

The following reagents were used for in vivo injections: LPS (Sigma, St. Louis, MO, USA), IL‐1β (Cell Signaling Technology, Danvers, MA, USA), and TNFα (R&D Systems, Minneapolis, MN, USA). PTH (1–84) used for the in vivo injections was purchased from Bachem California Inc. (Torrance, CA, USA). For CRISPR‐Cas9‐mediated production of the murine model, Cas9 protein was purchased from PNA Bio (Newbury Park, CA, USA). For diet‐induced CKD model, 0.67% Na Oxalate Diet (<0.01% Ca, 0.4% P; Cat. No. TD.110105) and Control Diet (0.6% Ca, 0.4% P; Cat. No. TD.97191) were purchased from Envigo (Indianapolis, IN).

### Generation of mutant mice

CRISPR mice were generated at the University of Wisconsin Biotechnology Center Transgenic Animal Facility. The guides used for CRISPR‐Cas9‐mediated genome editing were optimized for the least number of potential off‐target sites and fewest sites within coding exons using the Zhang Laboratory CRISPR Design tool (http://crispr.mit.edu). The following guides: m *Fgf23* −16kb Guide 1 GGAGGCGTAAACATCTGATC‐AGG (oligos F 5′‐CACC‐GGAGGCGTAAACATCTGATC‐3′ and R 5′‐AAAC‐GATCAGATGTTTACGCCTCC‐3′) and m *Fgf23* −16kb Guide 2 CCATGGGCTAGGGTGCGGAA‐TGG (oligos F 5′‐CACC‐GCCATGGGCTAGGGTGCGGAA‐3′ and R 5′‐AAAC‐TTCCGCACCCTAGCCCATGGC‐3′) were annealed and cloned into plasmids pX330 or pX458 obtained from the Zhang Laboratory via Addgene (Cambridge, MA, USA) as recently described.^35^, ^36^ RNA guides to be transcribed were produced by PCR amplification of the “target guide sequence and tracrRNA sequence” included in the pX330 and pX458 plasmids with primers containing the T7 promoter sequence (5′‐TTAATACGACTCACTATAGG‐) appended to the 5′ end of the original guide sequences with reverse primer 5′‐CAAAAAAGCACCGACTCGGTGCCACTTTTTCAAG‐3′, thus producing DNA PCR template of “T7 promoter − guide sequence − tracrRNA sequence.” These PCR products were then in vitro transcribed utilizing T7 MEGAshortscript (Life Technologies, Grand Island, NY, USA) kit.^37^ A mixture of 50 ng/μL of the produced RNA guides and 40 ng/μL of Cas9 protein in injection buffer (5 mM Trizma Base, 5 mM Tris HCL, 0.1 mM EDTA, pH 7.4) was injected into the pronucleus of 1 day fertilized embryos isolated from hyperovulating female C57Bl6 mice as described previously^38^ and implanted into recipient females. The resultant pups were genotyped with spanning primers, cloned, and sequenced for validation of the deletion. One mouse that contained the desired deletion of the −16kb region was identified (Fig.  2*B*) and was bred to produce the murine strain lacking the −16kb region. From here on, we will refer to this genetically modified mouse strain as the *Fgf23* ^−16KO^ mouse model.

### Animal studies

Mice were housed in high‐density ventilated caging in the Animal Research Facility of University of Wisconsin‐Madison under 12‐hour light/dark cycles, 72°F temperature, and 45% humidity. To induce FGF23 expression in vivo, animals were injected intraperitoneally (ip) with 10 mg/kg of body weight (bw) LPS, 2 μg recombinant TNFα, 50 ng/g bw recombinant IL1‐β, or phosphate‐buffered saline (PBS) as vehicle. These single doses are well within the range utilized by other investigators and were chosen to induce maximal response. Animals were euthanized and tissues collected 3 hours after Tnfα and 6 hours after IL‐1β or LPS injection. To test PTH regulation of FGF23 expression in vivo, animals were injected ip with 230 ng/g bw PTH (1–84) or vehicle (PBS) and euthanized for experiments 1 hour later. PTH and Tnfα effects on *Fgf23* transcription were previously determined by time‐course experiments to optimize for the effect examined (data not shown). For all the injections, the animals were stratified into groups according to their body weight. To induce CKD, 8‐week‐old mice were fed calcium‐deficient oxalate diet (0.67% Na Oxalate, <0.01% Ca, 0.4% P) or control diet (0.6% Ca, 0.4% P) for 7 days, at which time animals were euthanized and tissues collected. For each animal experiment, sex and age of the animals studied are indicated in each figure legend. All experiments and tissue collections were performed in the procedure rooms in the Research Animal Facility of University of Wisconsin‐Madison. All animal studies were reviewed and approved by the Research Animal Care and Use Committee of University of Wisconsin‐Madison.

### ChIP followed by sequencing (ChIP‐seq) and homology comparisons

In our prior work, we performed ChIP‐seq analysis on osteocyte‐differentiated IDG‐SW3 cells, as well as MSC‐derived osteoblasts and MC‐3T3‐E1‐ and UAMS‐derived osteoblasts.^35^, ^39^, ^40^ With regard to the IDG‐SW3 osteocyte line, cells were cultured for 35 days on collagen‐coated plates at 33°C with osteogenic media (αMEM with 10% heat‐inactivated FBS, 100 U/mL penicillin/streptomycin, 50 μg/mL ascorbic acid, and 4 mM glycerophosphate). Cells were then fixed with 1% formaldehyde and the calcified matrix was depleted through three 15‐minute 300 mM EDTA washes. ChIP‐seq analysis was performed in triplicate using a control antibody (IgG) or experimental antibodies (CTCF, H3K9ac, H4K5ac, H3K4me1, H3K4me2, and H3K27ac) as indicated.^41^ ChIP‐seq data processing and statistical evaluations was as described previously.^42^ All sequencing data have been publicly available in the Gene Expression Omnibus database: GSE54784.^41^ Epigenetic histone profiles obtained from MSCs, MC3T3‐E1, and UAMS cell lines were similar, suggesting enhancer profiles across the FGF23 gene locus are common within the mesenchymal cell lineage. Conservation analysis was performed with VISTA Point comparing mouse (mm9) to human (hg19) exclusively at the *FGF23* locus.^43^

### De novo transcription factor motif analysis

Genomatix MatInspector software was used for analysis of potential de novo transcription factor binding motifs. Matrix Library 8.4 versions of the transcription factor binding sites (Weight Matrices) were used. The −16kb region (chr6:127,006,796‐127,007,720) was evaluated for binding motifs of matrix families that have been suggested to play a role in inflammatory signaling (nuclear factor kappa B/c‐rel, hypoxia inducible factor‐ bHLH/PAS protein family members, and signal transducer and activator of transcription). Matrix information is supplied in Supplemental Table S1. The search was performed with a core similarity and optimal matrix similarity of 0.75 and only matches that exhibit a matrix similarity greater than 0.95 are shown in the Table 1.

**Table 1:** Transcription Factor Binding Motifs Identified by Genomatix MatIspector Analysis of *Fgf23* −16kb Element

Detailed matrix information	Strand	Core similarity	Matrix similarity	Sequence
Hypoxia‐response elements	–	1	0.985	tcttggtaCGTGagtgt
Binding site of Clock/BMAL1 and NPAS2/BMAL1 heterodimers	+	1	0.983	aagagtCACGtgtgctc
NF‐κB (p65)	–	1	0.974	ccaggactTTCCttt
STAT5	–	0.945	0.97	tagaTTCCtagaagtgaaa
STAT3	–	1	0.966	gctcTTCCagggaagtggt
AhR nuclear translocator homodimers	–	1	0.963	tgagcacaCGTGactct
STAT6	+	1	0.951	ccacTTCCctggaagagct

Bioinformatic analysis of the −16kb region using the Genomatix software tool MatInspector reveals the presence of several transcription factor binding motifs associated with inflammatory signaling and corresponding sequences along with highest calculated matrix similarity scores (greater than 0.95).

### Bone mineral density (BMD) analysis

At age 8 weeks, BMDs of *Fgf23* ^−16KO^ and their wild‐type littermates were measured and analyzed by dual‐energy X‐ray absorptiometry (DXA) with a PIXImus densitometer (GE Lunar Corp, Madison, WI, USA) as previously described.^30^

### Gene expression

Dissected tissues were frozen immediately in liquid nitrogen and stored at −80°C. Unless otherwise indicated, the bone gene expression analyses in this article were obtained from lumbar vertebra 5 (L_5_). Frozen tissues were homogenized in Trizol Reagent (Life Technologies) and RNA was isolated according to the manufacturer's instructions. One microgram of RNA was used as a template to synthesize cDNA using the High‐Capacity cDNA Reverse Transcription Kit (Applied Biosystems, Foster City, CA, USA). RNA isolation and cDNA production were performed in blinded fashion. Relative mRNA levels were determined via multiplex TaqMan quantitative reverse transcription‐PCR (RT‐PCR) using VIC‐labeled Mouse ACTB and FAM‐labeled TaqMan gene expression assays (Applied Biosystems). The following TaqMan Gene Expression probes (Applied Biosystems) were used for quantitative RT‐PCR: *Fgf23* (Mm00445621), *Il1b* (Mm00434228), *Il6* (Mm00446190), *Tnf* (Mm00443258), *Cyp27b1* (Mm01165918), *Cyp24a1* (Mm00487244), *Tnfsf11* (Mm00441906), *Fgf6* (Mm01183111), *Tigar* (Mm00621530), *Ccnd2* (Mm00438070), *Slc34a1* (Mm00441450), *Slc34a3* (Mm00551746), and mouse *Actb* (Cat. No. 4352341E). Relative mRNA levels were calculated using the ΔCt method.^30^

### Blood chemistry

Maxillary blood was collected during oxalate diet feeding at days 0, 4, and 7. For the remaining experiments, cardiac blood was collected at the time of death. Collected blood was incubated at room temperature for 30 minutes followed by duplicate centrifugation at 6000 rpm for 12 minutes to obtain serum or EDTA plasma. Serum calcium and phosphate levels were measured using QuantiChrom Calcium Assay Kit (Cat. No. DICA‐500, BioAssay Systems, Hayward, CA, USA) and QuantiChrom Phosphate Assay Kit (Cat. No. DIPI‐500, BioAssay Systems). Circulating intact FGF23 was measured in EDTA plasma via Mouse/Rat FGF‐23 (Intact) ELISA Kit (Cat. No. 60‐6800, Immutopics, San Clemente, CA, USA).

### Statistical evaluation

**Figure 1:**
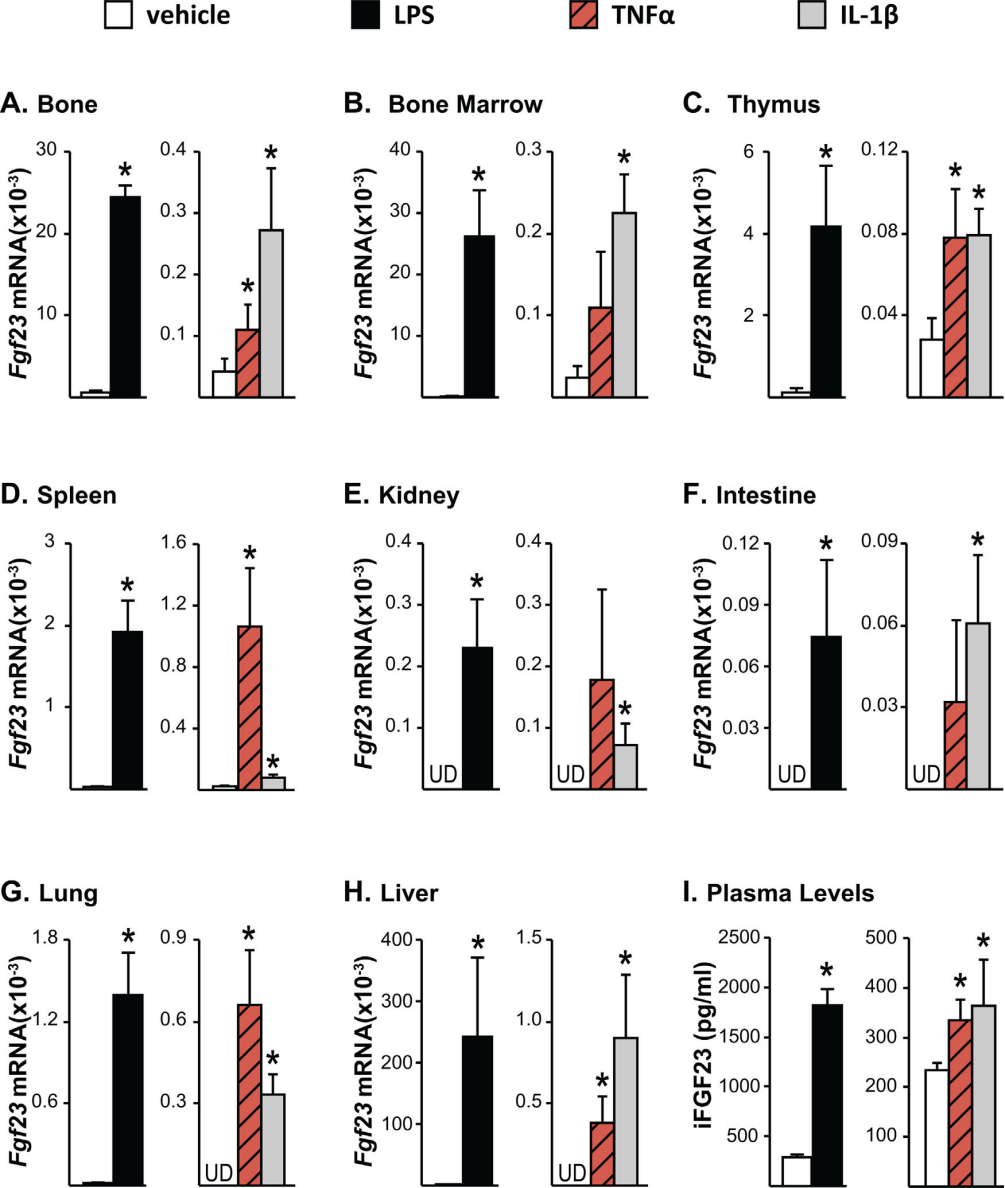
Inflammation increases *Fgf23* mRNA expression in bone and non‐osseous tissues. Twelve‐month‐old female mice were injected intraperitoneally (ip) with 10 mg/kg of LPS or PBS. Eight‐week‐old female mice were injected with 2 μg of recombinant TNFα, 50 ng/g of IL‐1β, or PBS. The tissues and blood were collected 3 hours after TNFα and 6 hours after LPS or IL‐1β injection. (*A–H*) *Fgf23* mRNA levels were measured by quantitative RT‐PCR and normalized to beta‐actin mRNA levels. (*I*) Blood was collected by cardiac puncture, and circulating intact FGF23 (iFGF23) levels were measured in EDTA plasma. (*A–I*) All values represent mean ± SD of 3 to 4 mice/group. All statistical comparisons were performed using Student's *t* test. *p < 0.05 compared with vehicle controls. UD indicates that *Fgf23* levels were undetectable by RT‐PCR.

All values represent the mean ± standard deviation (SD). Differences between group means were evaluated using Student's *t* test (Figs. 1 and 4) or one‐way ANOVA (Fig.  8 *A*) using GraphPad Prism 7 software (GraphPad Software, Inc., La Jolla, CA, USA). The data of Figs.  5, 6, 7, 8, 9 were analyzed by two‐way ANOVA with multiple comparison test using Benjamini‐Hochberg procedure as indicated in the figure legends. In two‐way ANOVA analysis of the data that did not pass normalization test in the original form, normalization was achieved by one of square root, log10, reciprocal, or rank transformations. If data did not achieve normality and equal variance after transformations, ranks were applied for nonparametric analysis. For two‐way ANOVA analysis of data obtained from tissues that normally do not express *Fgf23* mRNA, statistical analysis was performed by imputing minimum detectable levels in place of undetectable levels followed by permutation test analysis of a 2×2 ANOVA model.

## Results

### Acute inflammation induces *Ffg23* expression in multiple osseous and non‐osseous tissues

It has been previously shown that LPS induces *Fgf23* transcription in osteocytic cell lines and bone chips.^18^ However, *Fgf23* is expressed in non‐osseous tissues at low levels as well.^1^, ^20^, ^21^, ^22^ To address the scope of *Fgf23* regulation by acute inflammation in bone and non‐osseous tissues, we injected 8‐week‐old wild‐type (WT) mice with LPS (10 mg/kg bw). As indicated in Fig. 1 *A–D*, LPS increased *Fgf23* mRNA levels significantly in tissues that were shown previously to express *Fgf23* such as bone, bone marrow, thymus, and spleen. Interestingly, LPS injection also led to the enhanced expression of *Fgf23* in tissues such as kidney, intestine, lung, and liver that exhibit almost undetectable basal levels of *Fgf23* (Fig. 1 *E–H*).

Because LPS is known to significantly increase the production of multiple pro‐inflammatory cytokines such as IL‐1β, TNFα, and IL‐6 (as confirmed in Supplemental Fig. S1), LPS induction of *Fgf23* may be secondary to the actions of increased IL‐1β or TNFα. To explore this, we injected 8‐week‐old WT mice with recombinant IL‐1β (50 ng/g bw) or TNFα (2 μg). Although to a lesser extent when compared with LPS, both IL‐1β and TNFα induced *Fgf23* expression in all tissues examined (Fig. 1 *A–H*). In line with the *Fgf23* mRNA levels, IL‐1β and TNFα also increased circulating iFGF23 levels that were lower than those upregulated in response to LPS (Fig. 1 *I*). It is well recognized that LPS exerts effects that extend beyond those of an inflammatory nature. However, because the inflammatory cytokines show a similar response and because we see similar inductions of *Fgf23* mRNA levels in ex vivo calvarial cultures (data not shown), these results suggest that the predominant effect of LPS is to modulate *Fgf23* expression via its inflammatory effects, whether it be directly or in indirectly through the induction of additional inflammatory regulators. It is this latter mechanism wherein LPS induces multiple inflammatory cytokines that increase *Fgf23* in an additive fashion that may be responsible for the robust nature of LPS on *Fgf23* expression.

### A distal enhancer located 16kb upstream of *Fgf23* TSS mediates *Fgf23* expression

**Figure 2:**
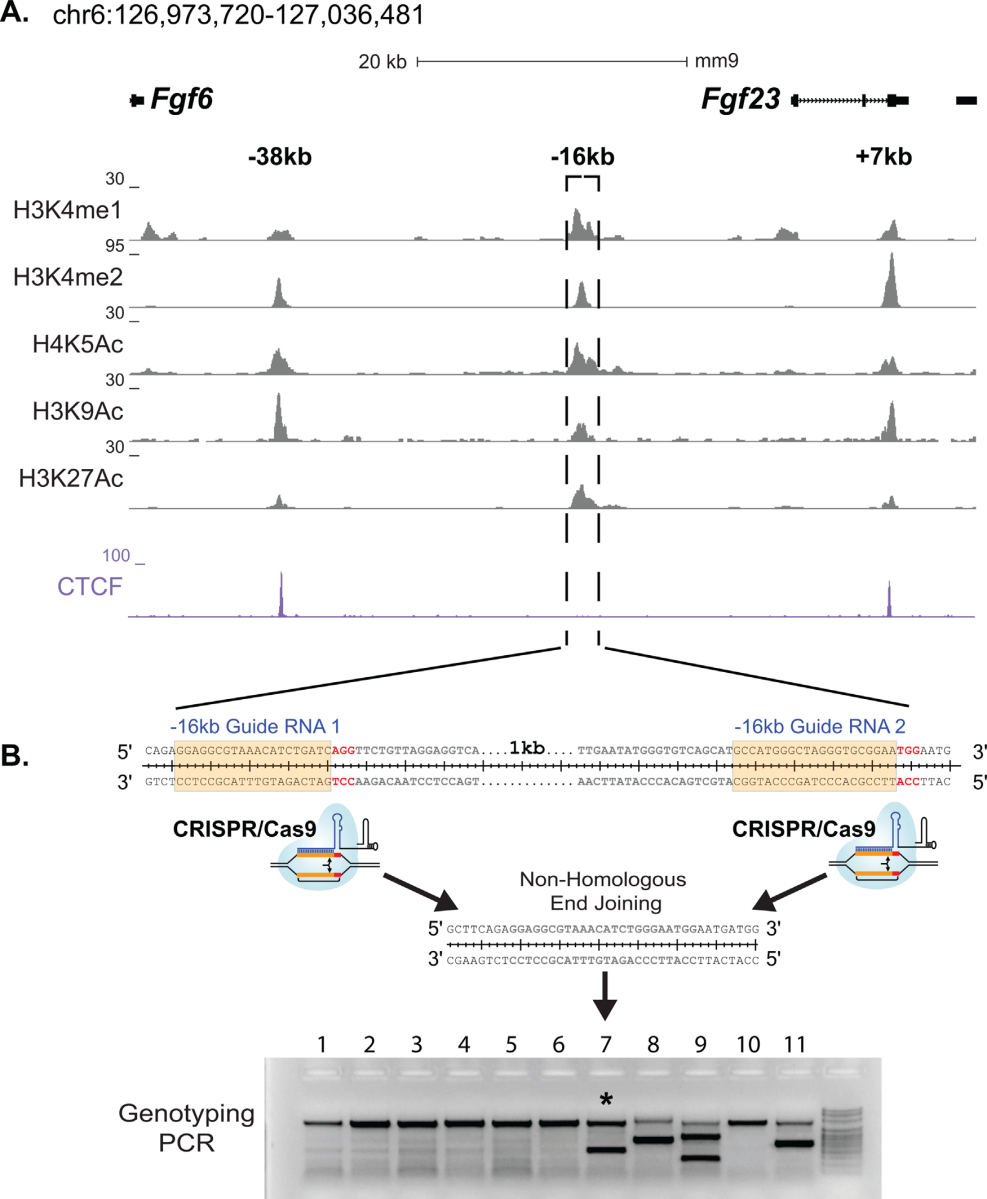
Identification of potential *Fgf23* enhancers and production of *Fgf23* ^−16KO^ mice. (*A*) ChIP‐seq tag density (normalized to 10^7^ tags) for histone modifications and CTCF binding across the *Fgf23* gene locus in differentiated IDG‐SW3 osteocytes. (*B*) Schematic describing the deletion of the −16kb region from the mouse genome via CRSIPR‐Cas9 genome editing.

**Figure 3:**
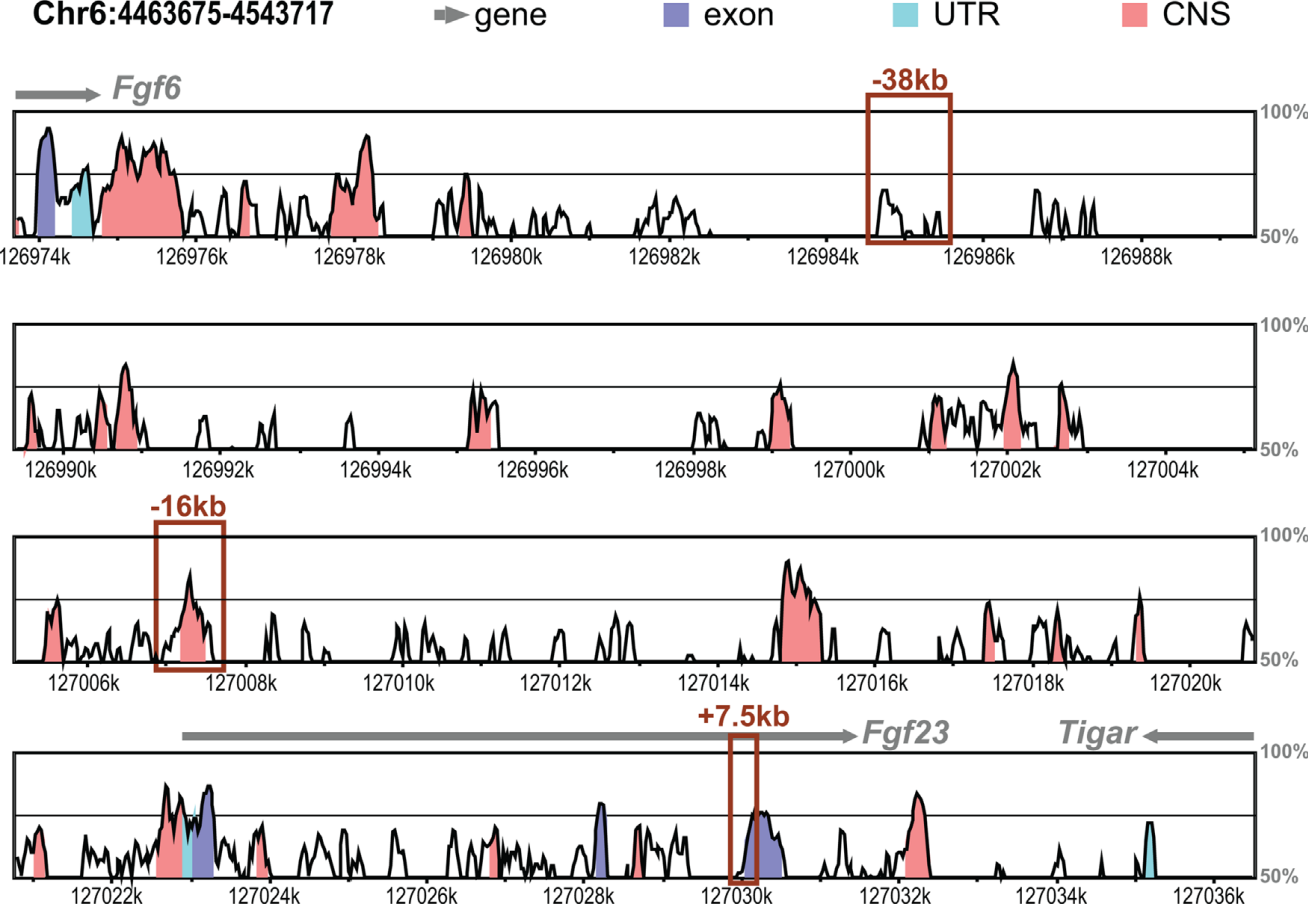
Comparative homology analysis of human and mouse *Fgf23* locus. Vista‐Point Tool was used for conservation analysis comparing the mouse (mm9) to human (hg19) *Fgf23* locus. Conservation score reaches significance at 70% (indicated by black middle line) for exons (dark blue), conserved untranslated region (UTR, cyan), and conserved noncoding sequence (CNS, coral). Other genomic features are indicated in legend. ChIP‐seq‐identified potential enhancers of *Fgf23* are shown with boxes.

We next sought to identify the transcriptional regulatory regions that mediate the induction of *Fgf23* in response to inflammatory stimuli. Inflammatory signaling involves multiple transcription factors including NF‐κB, HIF1α, signal transducers and activators of transcription (STATs), and perhaps others such as the nuclear receptor NURR1. Many of these have also been suggested to play a role in the regulation of *Fgf23*,^(18,19)^ although virtually all of these studies have focused on the promoter proximal region of the *Fgf23* gene and were conducted in vitro. Given this complexity, we took the alternative approach to examine data from our previous ChIP‐seq analysis of IDG‐SW3 osteocyte cells across the extended *Fgf23* gene locus in search of sites with overlapping H3K4me1, H3K4me2, H4K5ac, H3K9ac, and H3K27ac enrichment. These posttranslational histone marks are considered indicative of active regulatory regions and have been used by us previously to identify active regions in the *Tnfsf11* 44, 45 and *Mmp13* genes.^35^, ^36^ This search yielded three potential enhancers of *Fgf23* located −38kb, −16kb, and +7kb of the *Fgf23* TSS (Fig. 2 *A*), two of which (‐38kb and +7kb) were also occupied by CTCF (Fig. 2 *A*), a factor with multiple pleiotropic regulatory roles. We therefore focused on the −16kb region because this segment was also highly conserved between human and mouse (Fig. 3). Previous studies utilizing pathway inhibitors have shown that HIF1α and NF‐κB play a role in inflammatory induction of *Fgf23*. Bioinformatics analysis of these factors and STATs and showed that the −16kb region contained an array of in silico motifs for transcription factors involved in inflammation‐induced transcription (Table 1). Modest H3K4me1 activity was also found at the promoter region of *Fgf23*, suggesting potential regulatory activity within this region in vivo as well.

**Figure 4:**
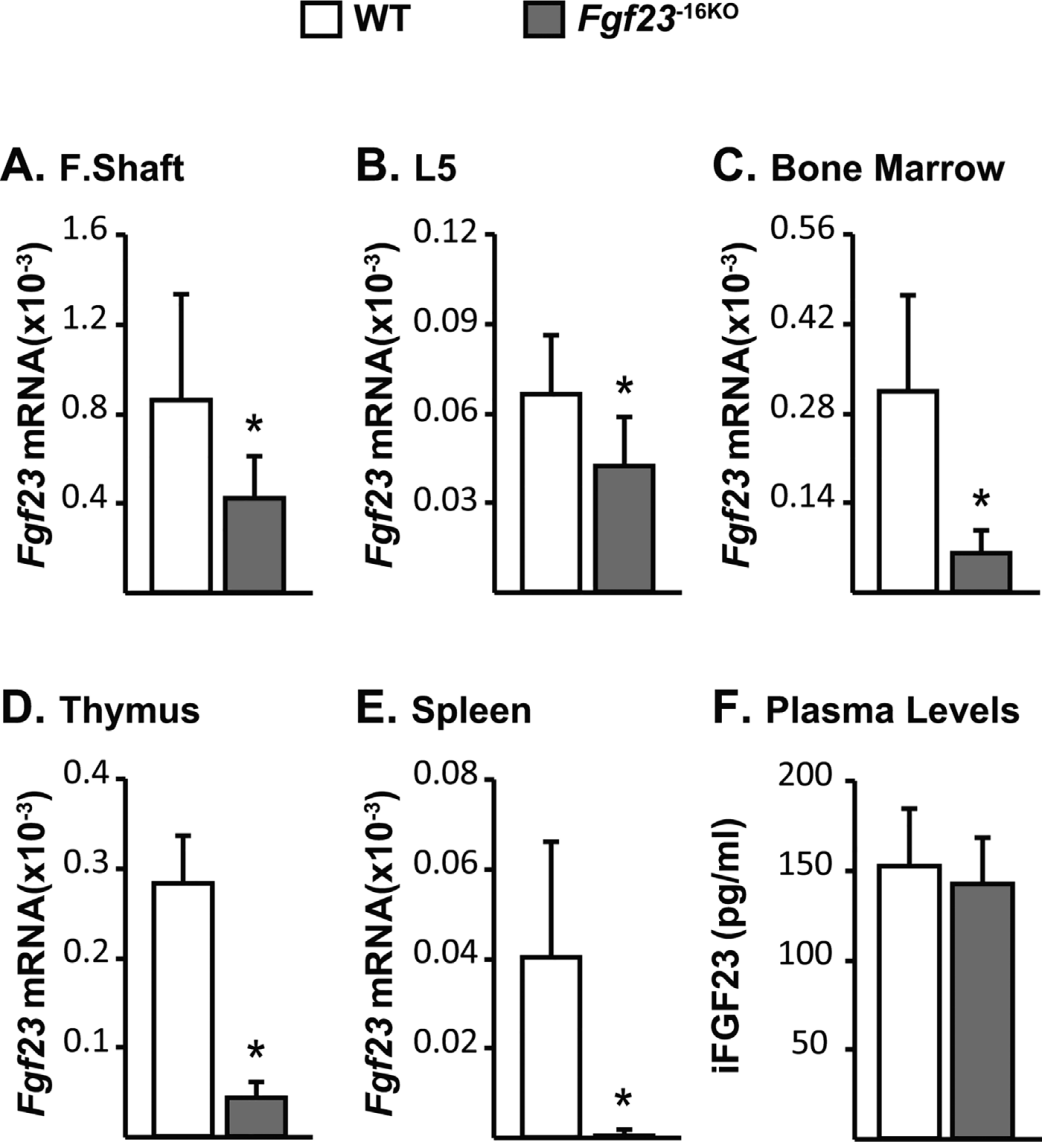
Lack of the −16kb region alters *Fgf23* mRNA levels but does not alter circulating iFGF23 levels. Tissues and cardiac blood of wild‐type (WT) and *Fgf23* ^−16KO^ male mice were collected at 8 weeks of age. (*A–E*) Femur shaft (F. Shaft) (*A*), lumbar vertebra 5 (L_5_) (*B*), and non‐osseous tissue (*C–E*) *Fgf23* mRNA levels were measured by quantitative RT‐PCR and normalized to beta‐actin mRNA levels. (*F*) Blood was collected by cardiac puncture, and circulating intact FGF23 (iFGF23) levels were measured in EDTA plasma. (*A–F*) Bars represent mean ± SD of 6 to 8 mice/group. All statistical comparisons were performed using Student's *t* test. *p < 0.05 compared with WT controls.

To determine if the potential enhancer at −16kb mediated *Fgf23* transcription, we deleted this region from the mouse genome utilizing the CRISPR‐Cas9 genome editing technique (Fig. 2 *B*) and created the *Fgf23* ^−16KO^ enhancer‐deleted mouse. Lack of the 16kb segment led to a ∼40% decrease in *Fgf23* mRNA levels in femur shafts and whole bones (Fig. 4 *A*, *B*) and to a more than 80% decrease in *Fgf23* mRNA levels in bone marrow, thymus, and spleen (Fig. 4 *C–E*). Moreover, deletion of the −16kb region did not alter the expression of the neighboring genes *Fgf6, Tigar*, or *Ccnd2* (Supplemental Fig. S2), suggesting that the −16kb region likely represents a bona fide enhancer that is linked directly to the regulation of *Fgf23* in vivo.

Despite the lower *Fgf23* mRNA levels, circulating iFGF23 levels of *Fgf23* ^−16KO^ mice were not different from their littermate controls (Fig. 4 *F*). This lack of change was supported by the observation that both *Fgf23* ^−16KO^ mice and their WT littermates displayed similar levels of expression of the renal FGF23 target genes that encode sodium‐dependent phosphate co‐transporter genes and the enzymes involved in 1,25(OH)_2_D_3_ metabolism (Supplemental Fig. S3). Similarly, *Fgf23* ^−16KO^ mice also maintain normal levels of serum calcium and phosphate, PTH levels, and bone mineral density (BMD) relative to their WT controls (Table 2). Taken together, these data suggest that lower *Fgf23* mRNA levels observed in *Fgf23* ^−16KO^ mice did not alter the overall biological activity of FGF23 in the blood. It is unclear why basal iFGF23 levels in the blood remain unchanged upon deletion of the −16kb *Fgf23* enhancer, although there are a number of examples of this phenomenon. It is worth noting that the basal levels of *Fgf23* are not only variable across the tissues examined but also extremely low and in some cases almost undetectable in non‐osseous tissues. Among all FGF23‐expressing tissues, bone expresses the highest basal amounts of FGF23 and the effect of the −16kb enhancer deletion in bone FGF23 expression is less compared with that in the non‐osseous tissues. Thus, it is possible that the impact on blood iFGF23 levels is cumulatively lost. However, tissue size is also of importance. It is also well known that FGF23 is dynamically processed posttranslationally through the complex actions of several different enzymes including Furin and Galnt 3 and that the differential activation of this processing event in tissues may be responsible for the discordance between mRNA and circulating iFGF23 levels. Further investigation of this phenomenon, however, is outside the objectives of this study of the transcriptional regulation of *Fgf23*.

**Table 2:** Bone and Mineral Homeostasis in *Fgf23* ^−16KO^ Mice

	Calcium (mg/dL)	Phosphate (mM)	PTH (pg/mL)
WT	10.7 ± 0.7	2.2 ± 0.5	55.2 ± 32.1
*Fgf23* ^−16KO^	10.3 ± 0.8	2.3 ± 0.3	50.9 ± 17.8

	Bone Mineral Density (BMD)		
	Femur	Spine	Global
WT	0.060 ± 0.005	0.045 ± 0.001	0.044 ± 0.002
*Fgf23* ^−16KO^	0.058 ± 0.005	0.046 ± 0.002	0.043 ± 0.002

Analysis of serum calcium and phosphate and plasma PTH were performed on 8‐week‐old wild‐type (WT) and *Fgf23* ^−16KO^ male mice as explained in Materials and Methods. BMD of these mice were measured by DXA. The values represent mean ± SD of 6 to 8 mice/group. All statistical comparisons were performed using Student's *t* test. *p < 0.05 compared with WT controls.

### The −16kb enhancer of *Fgf23* mediates inflammatory induction of *Fgf23*

**Figure 5:**
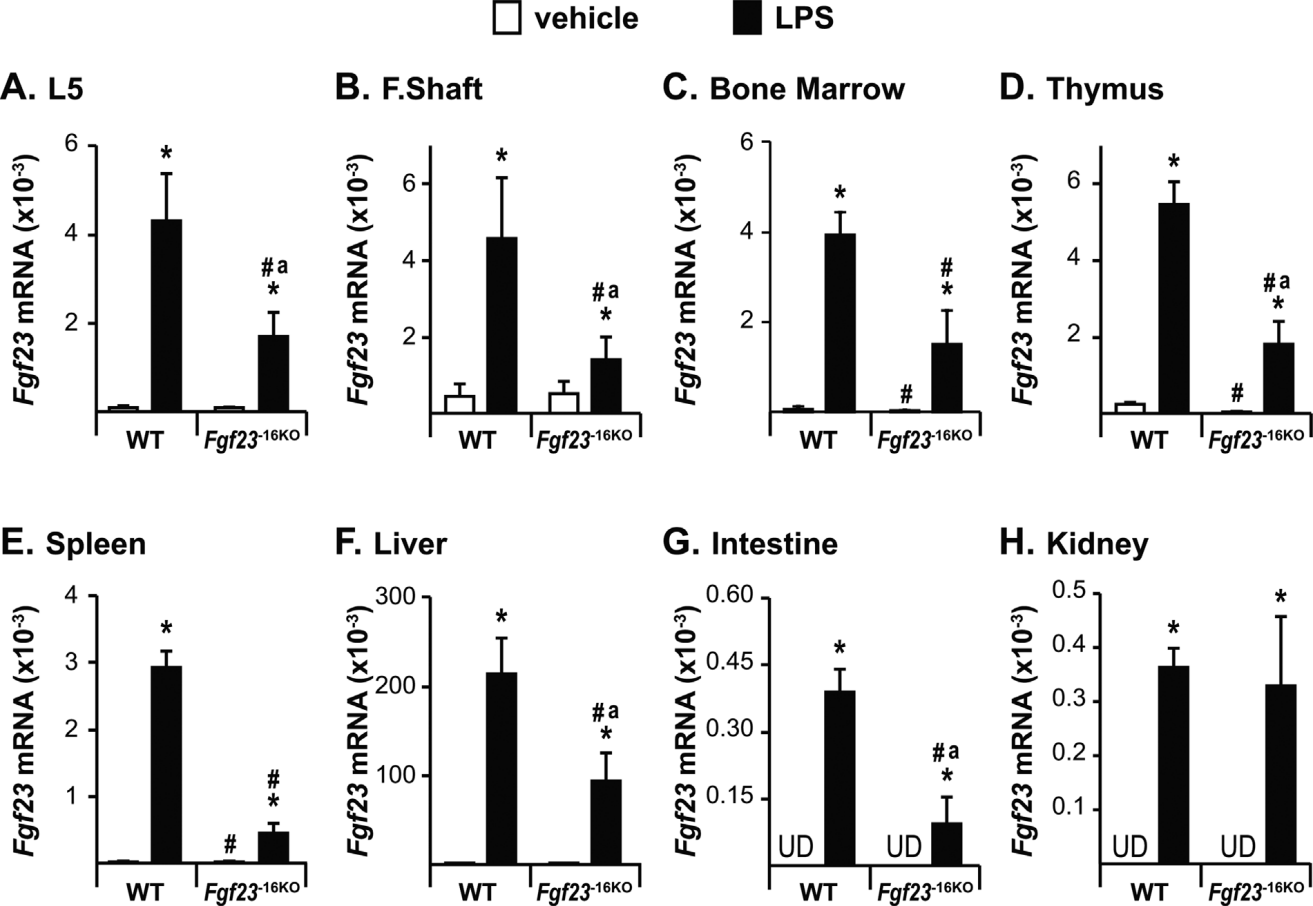
Lack of the −16kb enhancer of *Fgf23* blunts the LPS‐induced increase in *Fgf23* levels. Twelve‐week‐old wild‐type (WT) and *Fgf23* ^−16KO^ female mice were injected with 10 mg/kg of LPS or vehicle (PBS). Tissues were collected 6 hours after injection. (*A–H*) Lumbar vertebra 5 (L_5_) (*A*), femur shaft (F. Shaft) (*B*), and non‐osseous tissue (*C–H*) *Fgf23* mRNA levels were measured by RT‐PCR. Bars represent the mean ± SD of 4 to 6 mice/group. Statistical comparisons were performed using Two‐Way ANOVA with multiple comparison test using the Benjamini‐Hochberg procedure. *, *p*<0.05 effect of treatment within the same genotype; #, *p*<0.05 effect of genotype within the same treatment, and “a”, *p*<0.05 magnitude of the difference (vehicle‐injection) is less in *Fgf23* ^−16KO^ mice compared to WT mice. UD indicates that *Fgf23* levels were undetectable by RT‐PCR.

To assess the role of the −16kb *Fgf23* enhancer further, we injected *Fgf23* ^−16KO^ mice and their WT littermates with LPS. LPS injection induced IL‐1β, TNF, and IL‐6 expression similarly in both genotypes (Supplemental Fig. S1), suggesting equivalent inflammatory response in both genotypes. Similar to our observations shown in Fig. 1, LPS injection led to a significant induction of *Fgf23* mRNA in all tissues examined (Fig. 5 *A–H*). Lack of the −16kb *Fgf23* enhancer, however, blunted the LPS‐induced increase in *Fgf23* mRNA levels in bone, bone marrow, thymus, spleen, liver, and intestine (Fig. 5 *A–G*) but did not alter *Fgf23* induction by LPS in kidney (Fig. 5 *H*). These results suggest that this inflammatory modulator plays a distinct but perhaps tissue‐differential role at the genomic level in the transcriptional regulation of *Fgf23*.

**Figure 6:**
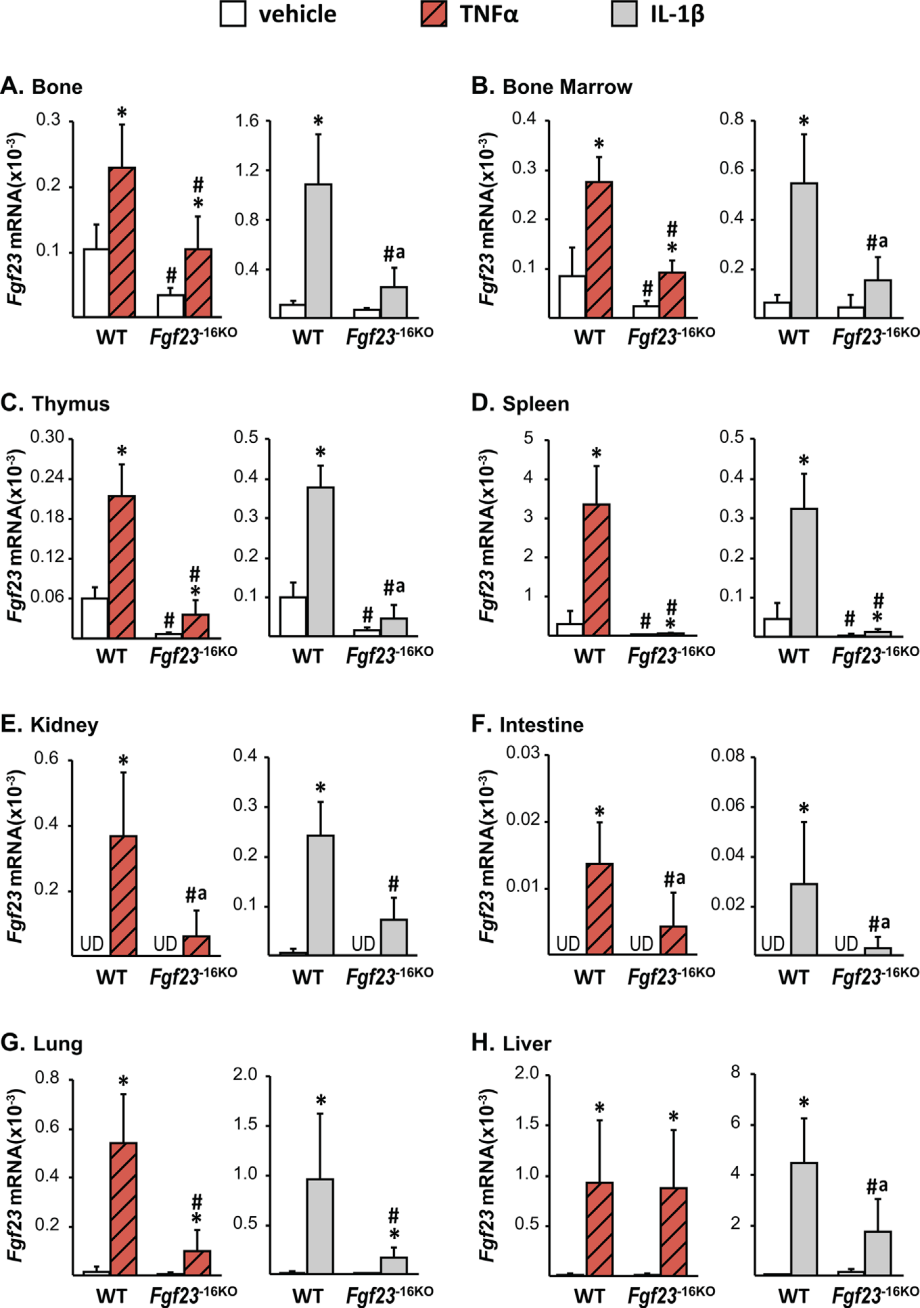
Lack of the −16kb enhancer of *Fgf23* blunts the Tnfα‐ and IL‐1β‐induced increase in *Fgf23* levels. Eight‐week‐old wild‐type (WT) and *Fgf23* ^−16KO^ female mice were injected with 2 μg of recombinant Tnfα or vehicle, while their male littermates were injected with 50 ng/g of IL‐1β or PBS. The tissues were collected 3 hours after TNFα and 6 hours after IL‐1β injection. The *Fgf23* mRNA levels were measured by RT‐PCR and represented here as the mean ± SD (*n* = 5 to 9 mice/group). Statistical comparisons were performed using two‐way ANOVA with multiple comparison test using the Benjamini‐Hochberg procedure. *p < 0.05 effect of treatment within the same genotype; #*p* < 0.05 effect of genotype within the same treatment, and “a”, *p* < 0.05 magnitude of the difference (vehicle‐injection) is less in *Fgf23* ^−16KO^ mice compared with WT mice. UD indicates that *Fgf23* levels were undetectable by RT‐PCR.

To determine whether the −16kb enhancer provided tissue‐specific regulation of *Fgf23* among the inflammatory cytokines, we injected *Fgf23* ^−16KO^ mice and their WT littermates with recombinant TNFα or IL‐1β. Both cytokines increased control gene expression similarly in all genotypes, confirming equivalent injection and inflammatory response (Supplemental Fig. S4). Lack of the −16kb enhancer blunted TNFα and IL‐1β induction of *Fgf23* in bone, bone marrow, thymus, spleen, kidney, intestine, and lung (Fig. 6 *A–G*). However, induction of *Fgf23* transcription by IL‐1β was different from that of TNFα in liver. Liver *Fgf23* mRNA levels increased to a lesser extent in *Fgf23* ^−16KO^ mice injected with IL‐1β compared with their littermate controls, suggesting control by the −16kb enhancer. In contrast, TNFα was fully active for *Fgf23* induction in the liver in *Fgf23* ^−16KO^ mice, suggesting that alternative regulatory elements or factors may be involved in *Fgf23* transcription in this organ. Moreover, although lack of the −16kb *Fgf23* enhancer did not alter LPS induction of *Fgf23* in kidney (Fig. 5 *H*), it blunted TNFα and IL‐1β induction of renal *Fgf23* production (Fig. 6 *E*), suggesting that LPS upregulation may be mediated via IL‐6 operating though other transcriptional control regions for *Fgf23*. These results point directly to the role of additional factors such as IL‐1β and Tnfα in the LPS regulation of *Fgf23* and that their induction by LPS may underlie the more complex response to this inflammatory inducer and may account for differential tissue‐specific activities as suggested earlier.

**Figure 7:**
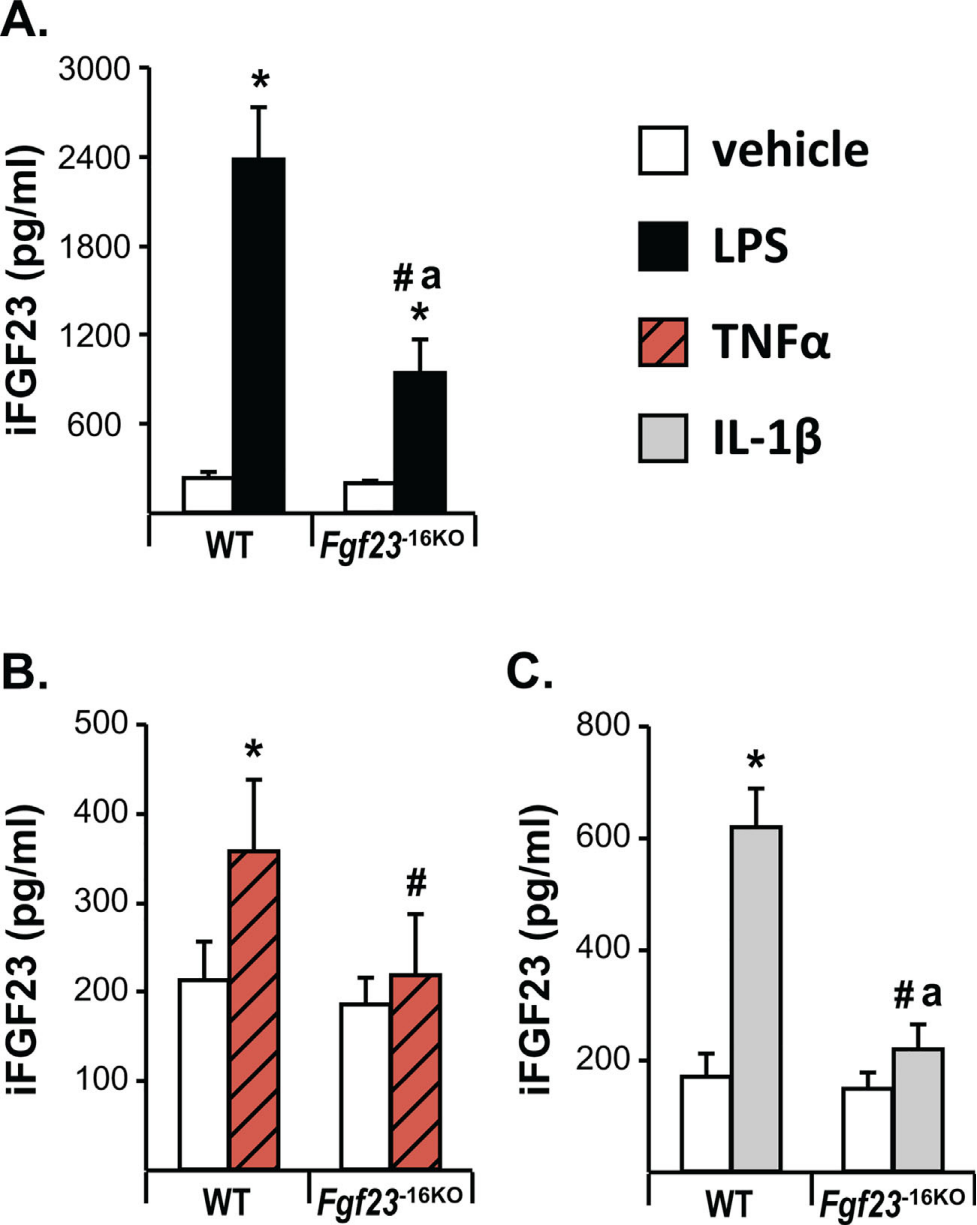
The −16kb enhancer of *Fgf23* mediates the inflammation‐induced increase in circulating intact FGF23 levels. Eight‐ to 12‐week‐old wild‐type (WT) and *Fgf23* ^−16KO^ mice were injected with 10 mg/kg of LPS (female mice) (*A*), 2 μg of recombinant TNFα (female mice) (*B*), 50 ng/g of IL‐1β (male mice) (*C*), or vehicle. Cardiac blood was collected at time of death, which was 6 hours after LPS and IL‐1β injection and 3 hours after TNFα injection. Circulating intact FGF23 (iFGF23) levels were measured by ELISA. The bars represent the mean ± SD of 4 to 9 mice/group. Statistical comparisons were performed using two‐way ANOVA with multiple comparison test using the Benjamini‐Hochberg procedure. *p < 0.05 effect of treatment within the same genotype; #*p* < 0.05 effect of genotype within the same treatment and “a”, *p* < 0.05 magnitude of the difference (vehicle injection) is less in *Fgf23* ^−16KO^ mice compared with WT mice.

Despite the fact that basal changes in *Fgf23* mRNA levels of *Fgf23* ^−16KO^ mice did not alter circulating iFGF23 levels (Fig. 4 *F*), lack of the −16kb enhancer significantly blunted, or in the case of TNFα injections prevented, the inflammation‐induced increases found in circulating iFGF23 levels (Fig. 7 *A–C*). As summarized in Table 3, these results collectively suggest that the −16kb enhancer mediates *Fgf23* expression in response to inflammation in a tissue‐specific manner. In contrast, lack of the −16kb region did not alter expression or transcriptional regulation of the nearby genes *Ccnd2* (data not shown), *Fgf6*, and *Tigar* (Supplemental Table S2), suggesting that transcriptional regulation mediated by the −16kb enhancer was restricted to the *Fgf23* gene.

**Table 3:** Summary of the Role of the −16kb Enhancer in *Fgf23* Regulation in Response to Acute Inflammation and Kidney Disease

	*Fgf23* mRNA levels								
	Bone	Bone marrow	Thymus	Spleen	Kidney	Intestine	Lung	Liver	iFGF23 Plasma
IL‐1β	+	+	+	+	+	+	+	+	+
Tnfα	+	+	+	+	+	+	+	–	+
LPS	+	+	+	+	–	+	+	+	+
Oxalate diet	+	UD	+	x	+	UD	UD	x	+

A comparison of the results of *Fgf23* mRNA regulation by inflammatory cytokines and CKD in different tissues with levels in the circulation as observed in Figs. 3, 4, 5, 6, 7.

+ = −16kb enhancer plays a role in the *Fgf23* induction; − = −16kb enhancer does not play a role in *Fgf23* induction; X = *Fgf23* is not regulated; UD = *Fgf23* levels were not measured.

### The −16kb distal enhancer of *Fgf23* mediates the early onset induction of *Fgf23* in a kidney disease model

**Figure 8:**
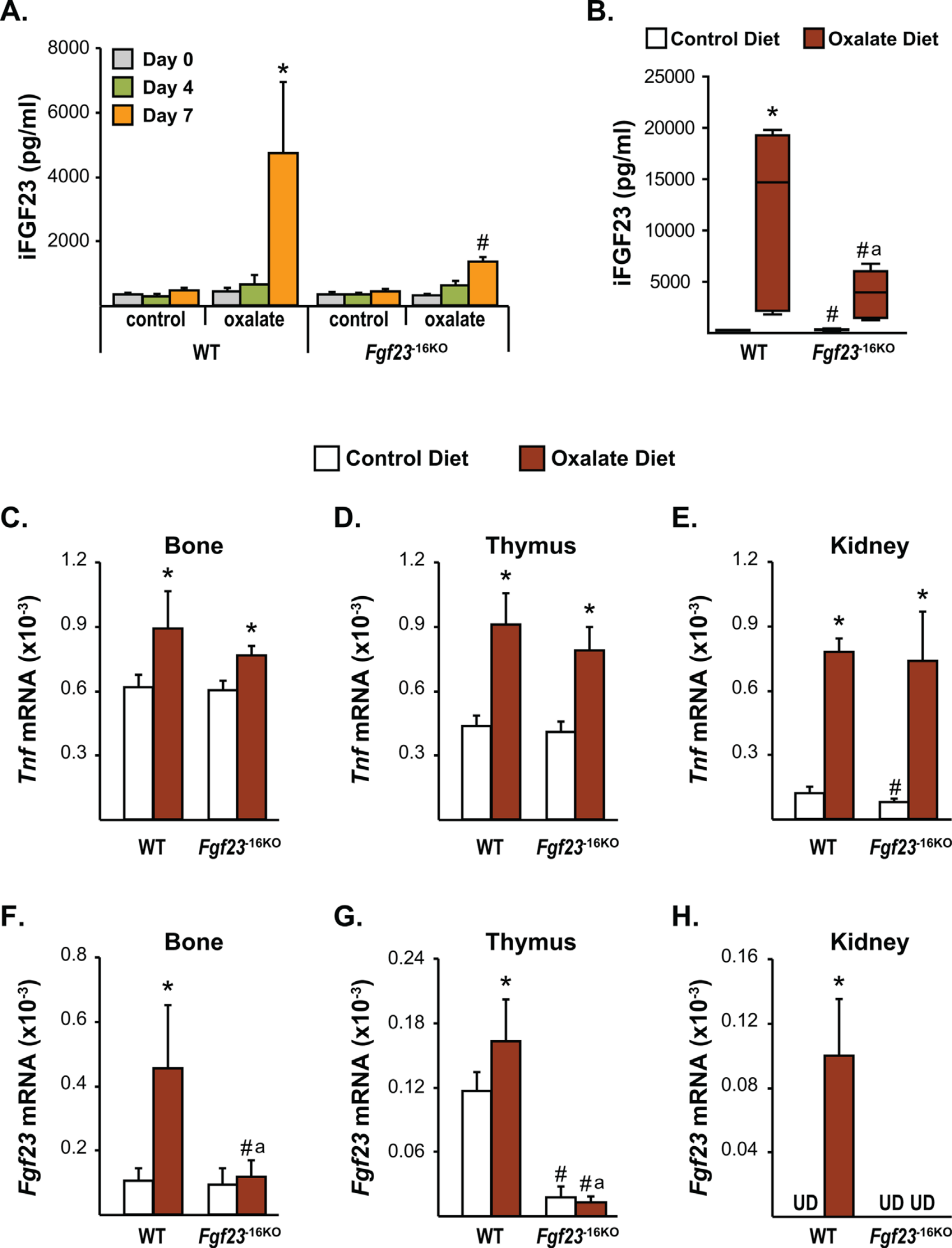
Lack of the −16kb enhancer of *Fgf23* prevents kidney disease‐induced increase in *Fgf23* levels. Eight‐week‐old female (*A*) and male (*B–H*) *Fgf23* ^−16KO^ mice and their wild‐type (WT) littermates were fed control or oxalate diet for 1 week. (*A*, *B*) Circulating intact FGF23 (iFGF23) levels were measured at day 0 (*A*), day 4 (*A*), and day 7 (*A*, *B*) of the diet feeding. (*C–H*) After 7 days of diet feeding, *Tnfα* (*C–E*) and *Fgf23* (*F–H*) mRNA levels of the male *Fgf23* ^−16KO^ and WT mice were measured by RT‐PCR. (*A*) Bars represent the mean ± SD of 3 to 6 mice/group and statistical analysis were performed using one‐way ANOVA. For this analysis, *p < 0.05 effect of oxalate diet compared with day 0 value of the same genotype; #*p* < 0.05 effect of genotype within the same diet at the same time point. (*B*) In this box and whisker plot of iFGF23, whiskers represent the range of the values, the line located in the boxes represent the median, and the boxes represent the second and third quartiles of 5 to 7 mice per group and the statistical analysis between the groups were performed using two‐way ANOVA with Tukey's multiple comparison test. (*C–H*) Bars represent the mean ± SD of 5 to 7 mice/group and statistical analysis were performed using two‐way ANOVA with multiple comparison test using the Benjamini‐Hochberg procedure. For all analysis of *B–H*, *p < 0.05 effect of diet within the same genotype; #*p* < 0.05 effect of genotype within the same diet and “a”, *p* < 0.05 magnitude of the difference (control diet‐oxalate diet) is less in *Fgf23* ^−16KO^ mice compared with WT mice. UD indicates that *Fgf23* levels were undetectable by RT‐PCR.

Recent studies have shown a positive correlation between elevations in inflammatory markers and FGF23 levels in CKD patients beginning at very early stages of the disease,^46^, ^47^ raising the possibility that inflammation may cause or contribute to the increase in FGF23 production in CKD. Because the −16kb enhancer mediates inflammatory regulation of *Fgf23* transcription, we next utilized a diet‐induced CKD model to address whether the −16kb enhancer could play a role in mediating the CKD‐induced increase in *Fgf23* expression.^48^ It has been shown previously that oxalate diet feeding initiates altered kidney function as early as 7 days post feeding, whereas 3 weeks of oxalate diet feeding is required to obtain the majority of features of CKD in this model.^48^ Although this is not a perfect model of CKD because of the rapid rather than chronic progression of the disease, it allowed us to test whether diet‐induced *Fgf23* upregulation was mediated by the −16kb enhancer. To determine the time‐course of iFGF23 induction in the oxalate feeding‐induced CKD, we fed 8‐week‐old female and male *Fgf23* ^−16KO^ mice and their littermate controls a control or oxalate diet and demonstrated that circulating iFGF23 increases after 7 days of oxalate diet feeding in WT mice (Fig. 8 *A*, *B*). Although 1 week of oxalate diet increased WT iFGF23 to a greater level in male mice, both female and male mice lacking the −16kb enhancer exhibited a blunted increase in circulating iFGF23 levels at this time point (Fig. 8 *A*, *B*).

In the genetically induced murine *Col4a3* CKD knock‐out model, increases in circulating iFGF23 levels have been shown to precede an increase in *Fgf23* mRNA levels in bone,^49^ suggesting the possibility that FGF23 production in non‐osseous tissues may contribute to the CKD‐induced increase in circulating iFGF23 levels. Because we observed that inflammation can induce *Fgf23* mRNA expression in multiple non‐osseous tissues, we next sought to determine potential sources of the CKD‐induced increase in iFGF23 levels and to assess whether the upregulation of these levels of iFGF23 were mediated by the −16kb enhancer of *Fgf2*3. Gene expression analysis performed 1 week after oxalate diet feeding showed similar increases in *Tnfα* expression of osseous and non‐osseous tissues of both WT and *Fgf23* ^−16KO^ mice, suggesting that the oxalate diet increased inflammation in both genotypes similarly (Fig. 8 *C–E*). In accordance with the increased circulating iFGF23 levels, 1 week of oxalate diet feeding of WT mice increased *Fgf23* mRNA levels in bones, thymus, and kidney (Fig. 8 *F–H*) but not in liver or spleen (data not shown). Despite a blunted, but significant, increase in iFGF23 levels (Fig. 8 *B*), *Fgf23* ^−16KO^ mice did not show a significant increase in *Fgf23* mRNA levels in any of the tissues examined (Fig. 8 *F–H*). Taken together, these results suggest that −16kb enhancer of *Fgf23* mediates the early onset of CKD‐induced increase in bone, thymus, and kidney *Fgf23* transcription but that this upregulation contributes only partially to changes in circulating iFGF23 levels.

### The −16kb distal enhancer of *Fgf23* mediates PTH‐induced increase in *Fgf23* transcription

**Figure 9:**
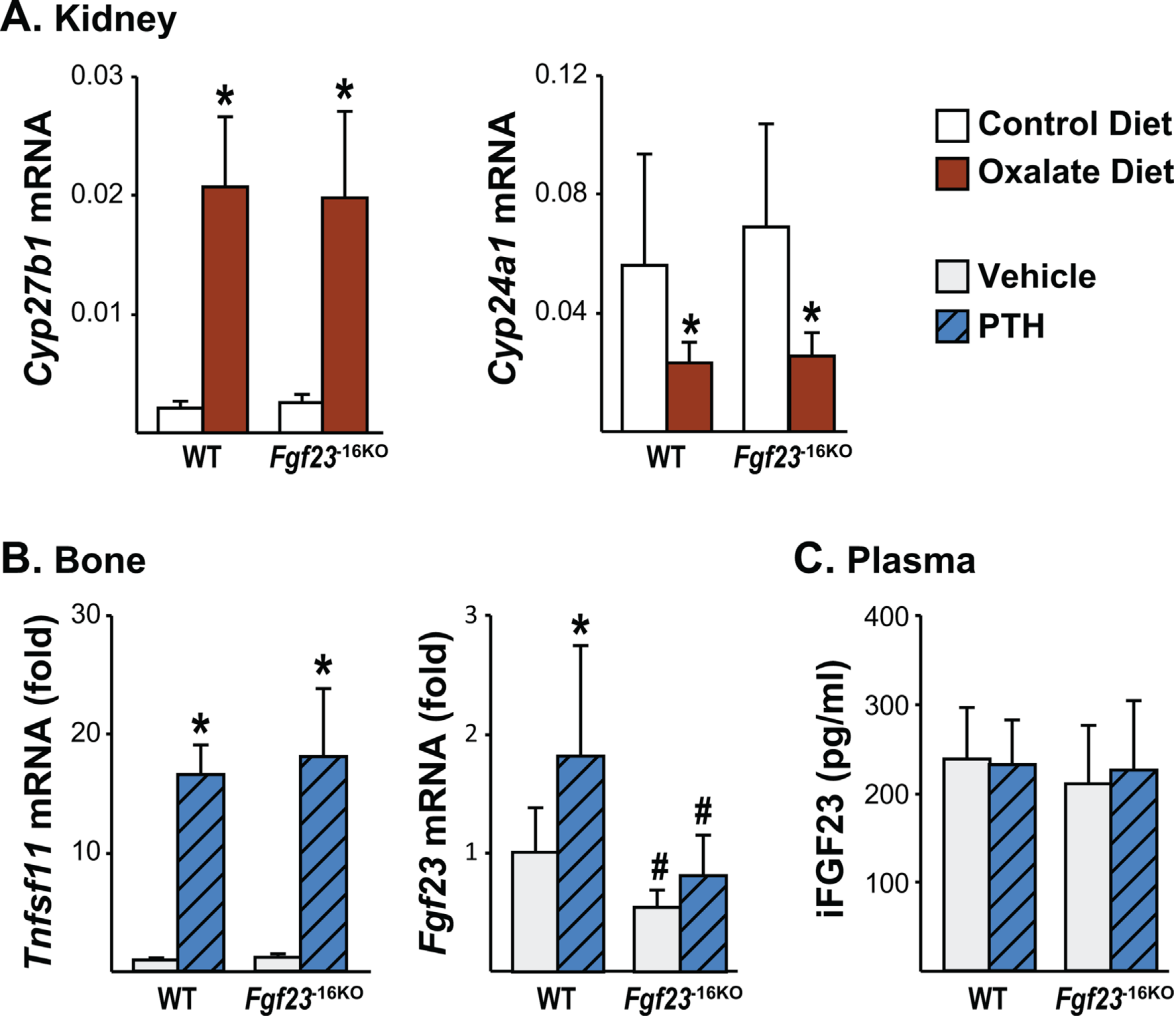
The −16kb enhancer of *Fgf23* mediates the PTH‐induced increase in *Fgf23* transcription. (*A*) Eight‐week‐old male *Fgf23* ^−16KO^ mice and their wild‐type (WT) littermates were fed control or oxalate diet for 1 week. Kidney *Cyp27b1* and *Cyp24a1* mRNA levels were measured by RT‐PCR. Bars represent the mean ± SD of 5 to 7 mice/group. (*B*, *C*) Eight‐week‐old wild‐type (WT) and *Fgf23* ^−16KO^ male mice were injected ip with 230 ng/g bw of PTH. Tissues were collected 1 hour after injection. (*B*) *Tnfsf11* (RANKL) and *Fgf23* mRNA levels were measured by RT‐PCR. Bars represent the mean ± SD of 8 to 9 mice/group as fold by comparison to mean of WT vehicle group. (*C*) Circulating intact FGF23 (iFGF23) levels were measured 1 hour after PTH injection. Bars represent the mean ± SD of 8 to 9 mice/group. All statistical analysis between the groups were performed using two‐way ANOVA with multiple comparison test using the Benjamini‐Hochberg procedure and “a”, *p* < 0.05 magnitude of the difference (vehicle‐PTH injection) is less in *Fgf23* ^−16KO^ mice compared with WT mice. *p < 0.05 effect of treatment within the same genotype; #*p* < 0.05 effect of genotype within same treatment.

In addition to inflammation, PTH levels also progressively increase during CKD. Indeed, high PTH levels have also been proposed to induce *Fgf23* transcription.^(50)^ Consistent with this concept, 1 week of oxalate diet increased PTH levels, as indicated by the increase in *Cyp27b1* and decrease in *Cyp24a1* mRNA levels (Fig. 9 *A*). PTH‐PTH 1 Receptor (PTH1R) signaling activates the protein kinase A (PKA) pathway, among others, that leads to the phosphorylation of cAMP response element binding protein (CREB) followed by phospho‐CREB (pCREB) translocation to the nucleus, where it drives PTH‐mediated gene expression. However, previous in vitro studies utilizing the UMR106 cell line have suggested that PTH upregulates *Fgf23* transcription and that this action may be secondary to and mediated by induction of the orphan nuclear receptor NURR1.^51^ Although we did not observe pCREB binding in response to PTH in the −16kb region in our previous ChIP‐seq analysis,^39^ there are NURR1 binding motifs in the −16kb region. To test whether PTH mediates *Fgf23* expression via the −16kb region, we injected *Fgf23* ^−16KO^ mice and their WT littermates with 230 ng/g bw PTH. As expected, PTH increased *Tnfsf11* (RANKL) mRNA levels similarly in both genotypes, confirming equivalent treatment of all mice (Fig. 9 *B*). WT mice injected with PTH showed a twofold increase in *Fgf23* mRNA levels in bones (Fig. 9 *B*) but not in other tissues such as kidney (data not shown). Moreover, lack of the −16kb distal enhancer of *Fgf23* prevented this PTH‐induced increase in *Fgf23* mRNA levels (Fig. 9 *B*). However, in line with a recent report from Knab and colleagues,^52^ in our experiment acute single PTH injection did not increase plasma iFGF23 levels in WT or *Fgf23* ^−16KO^ mice (Fig. 9 *C*), providing another example of discordance between transcriptional regulation and processing. Collectively, our results suggest that although both PTH and inflammation mediate bone *Fgf23* transcription via the −16kb region, the effects of PTH on *Fgf23* levels are restricted to bone and are much milder compared with that of inflammation.

## Discussion

Previous studies have shown that inflammation and calciotropic hormones increase *Fgf23* transcription.^18^, ^21^, ^23^, ^51^, ^52^, ^53^, ^54^ However, the molecular mechanisms underpinning the transcriptional regulation of *Fgf23* in response to inflammation and PTH in bone or non‐osseous tissues are unknown. Herein, we utilized unbiased genome‐wide analysis of epigenetic marks to identify several potential enhancers for *Fgf23* and by deletion of this enhancer in vivo showed that a distal enhancer located −16kb upstream of the *Fgf23* gene's TSS mediates basal‐, inflammation‐, and PTH‐induced expression of *Fgf23* in a tissue‐specific manner. Because both inflammation and PTH are suggested to mediate FGF23 levels in CKD, we employed a diet‐induced CKD model and showed that the −16kb enhancer mediates induction of *Fgf23* in thymus, kidney, and bone and that this led to a blunting of the increase in iFGF23 levels in circulation at early onset of this kidney disease model. Collectively, our findings support a complex role of the −16 kb *Fgf23* enhancer in mediating inflammatory and other effects of *Fgf23* regulation.

Importantly, this study describes the initial phases of a new approach whereby the locations of potential enhancers are identified first through unbiased genetic and/or epigenetic ChIP‐seq analyses and then explored subsequently through loss‐of‐function studies directly in the mouse in vivo. The advantages of this approach are extensive, as they enabled us to 1) link putative enhancers directly to their target genes via genomic and RNA output activity in vivo; 2) establish primary functions for enhancers; 3) identify new and unsuspected roles for individual enhancers; 4) assess activity across multiple tissues, thereby enabling the study of selective activity; and 5) avoid detailed analyses of mechanism (definition of transcription factor participation, activation, and upstream signaling processes) until such time as the regulatory regions are fully validated in vivo. This new approach is necessary because recent studies have now shown that most genes are regulated by multiple enhancers, many of which are located at sites distal to the genes they regulate and because promoter proximal studies utilized for the past several decades are now known to be highly biased and more frequently unreliable. As outlined here, we have now established that the −16kb enhancer represents a bone fide regulatory segment of the *Fgf23* gene and that at least one of its activities is to facilitate the upregulatory activity of inflammatory mediators such as LPS, IL‐1β, and Tnfα, as well as the calciotropic hormone PTH at multiple tissues, many of which were not suspected to contribute to FGF23 production until now. This approach has also enabled us to define a disease role for the −16kb enhancer as a mediator of *Fgf23* upregulation in a mouse model of the early stages of CKD. The results hint at a direct primary role for inflammation in that upregulation, but more importantly, now provide an experimental approach for defining the molecular details of that upregulation in vivo. Further studies are now focused on the activities of the additional putative regulatory regions within and surrounding the *Fgf23* gene to understand their potential roles in mediating the activities of additional systemic regulators such as phosphate and 1,25(OH)_2_D_3_. Once these are established, studies can focus on the mechanistic details that relate to the identities of the transcription factors that are involved, the recruitment of epigenetic factors that may be involved in modulating *Fgf23* output, and delineation of the upstream signaling pathways that transduce environmental information as to the physiologic or pathophysiologic state of the organism.

Because the highest concentration of FGF23 is produced by osteocytes, the regulation of FGF23 synthesized in non‐osseous tissues has been largely unexplored. However, elevations in the non‐osseous expression of *Fgf23* have been observed in multiple diseases and in animal models of disease. For example, studies by Zanchi and colleagues have recently shown increases in renal *Fgf23* expression in a model of progressive kidney injury induced by diabetes.^55^ In more recent studies, Bansal and colleagues and Wongdee and colleagues have shown elevations in splenic^23^ and intestinal^56^ expression of *Fgf23*. Herein, we provide additional evidence that acute inflammation can induce *Fgf23* expression in several non‐osseous tissues such as thymus, spleen, bone marrow, kidney, intestine, lung, and liver and show that *Fgf23* is increased even at early stages in the oxalate diet‐induced CKD model, not only in bone but also in non‐osseous tissues such as thymus and kidney. Moreover, although we show that both PTH and inflammation can increase *Fgf23* levels in bone, only inflammation appears capable of inducing non‐osseous *Fgf23* expression, suggesting that the higher *Fgf23* levels observed in CKD bones may be due to elevated PTH or inflammation, whereas the increased non‐osseous *Fgf23* levels are likely due to increased inflammation. Taken together, these results suggest that *Fgf23* elevations are not limited to osseous tissues in inflammatory disorders and point out the need for further research on the pathological roles of these non‐osseous sources of FGF23.

Osteocytes are the major source of FGF23 in bones under physiological conditions and in response to inflammatory stimuli.^18^, ^19^, ^57^ In contrast, macrophages^21^ and dendritic cells^21^, ^22^ have been suggested to be the main source of FGF23 in spleen and bone marrow under normal or inflammatory conditions. Because the majority of the tissues contain tissue‐resident macrophages that play many roles including iron processing and immune response,^58^ macrophages may represent a potential source of non‐osseous FGF23 induced by inflammation. Importantly, macrophages from various tissue sources are not identical, and indeed the properties of resident macrophages can take on genetic and epigenetic features as determined by the host tissue. Thus, whether the inflammation‐induced *Fgf23* elevations in non‐osseous tissues are due to migration of *Fgf23*‐expressing immune cells into these tissues or to induction of *Fgf23* expression in cell types residing in these tissues is unknown and will require further investigation.

Several studies have shown that *Fgf23* expression is mediated by inflammatory cytokines; however, the molecular mechanisms underpinning this regulation are unclear. Herein, we utilized ChIP‐seq analysis of epigenetic marks and identified the −16kb enhancer, which contains multiple binding motifs for transcription factors, including HIF1α, NF‐κB, and STATs that have been suggested to mediate *Fgf23* transcription in response to inflammation.^(18,19,21)^ Deletion of the −16kb enhancer of *Fgf23* significantly reduced but did not completely prevent inflammatory induction of *Fgf23* in bone or in other tissues, suggesting the presence of additional regulatory elements that control the output of this gene. Moreover, our results also show that the overall role of the −16kb region in controlling inflammation‐induced *Fgf23* expression may be different in some tissues. Although this is likely to be due to the differential interaction of residual transcription factors that bind this enhancer, it is possible that additional enhancers may be present in other tissues that impact this differential activity. ChIP‐seq analysis of additional cell lineage types is in progress to evaluate this possibility. Given the rather ubiquitous ability of inflammatory signals to regulate FGF23 expression, we would predict that the −16 kb enhancer will likely be present in most tissues.

Our previous studies have also shown that deletion of a single enhancer can alter the transcriptional contribution of other enhancers linked to the same gene.^35^ Thus, although we have observed a blunting of the inflammatory response of *Fgf23* expression in *Fgf23* ^−16KO^ mice and identified possible motifs for several candidate transcription factors at this site, we cannot rule out the possibility that the −16kb enhancer may also function by altering the activity of additional regulatory regions playing a role in *Fgf23* gene regulation. This type of interrelationship could also explain the variable contribution of the −16kb enhancer to *Fgf23* expression in different tissues, although this may also be due to the actions of LPS, IL‐1β, and/or Tnfα to modulate different collections of transcription factors at the *Fgf23* gene in the individual tissues. It is for these reasons that we anticipate delineating the activities of each of the additional regulatory regions of the *Fgf23* gene before conducting detailed ChIP‐seq analysis to identify the multiple transcription factor candidates that are likely to play roles in mediating these tissue‐specific physiological as well as inflammatory responses. As stated earlier, this represents a largely new paradigm for studying transcriptional regulation that is based upon functional validation of enhancers in tissues in vivo before in‐depth analysis of the transcription factors and signaling pathways that may be involved.

Both inflammation and PTH have been shown to increase *Fgf23* production^18^, ^19^, ^50^, ^52^ and therefore may be potential inducers of *Fgf23* expression in CKD. Herein, we show that lack of the −16kb enhancer of *Fgf23* prevented early onset kidney disease‐induced increases in renal, thymic, and skeletal *Fgf23* expression in a diet‐induced CKD model. Although the expression of inflammatory cytokines such as TNFα and IL‐1β are induced in multiple tissues in our oxalate diet‐induced disease model, circulating PTH was also increased. In the experimental setting of an acute single injection, inflammation increases both *Fgf23* mRNA levels and circulating iFGF23 in the blood at higher magnitudes compared with induction of *Fgf23* by a single injection of PTH. Nevertheless, PTH levels in CKD are much higher and are sustained for a longer duration compared with those resulting from an acute single PTH injection. They also occur in a setting of uremia; therefore, PTH cannot be ruled out as a potential regulator of the osseous production of *Fgf23* in CKD. Because both inflammation and PTH utilize the same −16kb region to mediate *Fgf23* transcription, further refined dissection of the −16kb enhancer will be necessary to identify which of these *Fgf23* mediators represents the primary inducer of FGF23 in CKD.

In summary, our data indicate that inflammation‐induced *Fgf23* expression is not limited to osseous tissues, and although induction of both skeletal and non‐osseous tissue sources of *Fgf23* is mediated via the −16kb distal enhancer, transcriptional regulation of *Fgf23* gene may also be controlled by additional enhancers operating in concert. Moreover, whereas PTH regulation of *Fgf23* levels is less robust compared with inflammatory regulation, it is also mediated by the −16kb region. Finally, transcriptional regulation of *Fgf23*, as exemplified here by the oxalate diet‐induced CKD model, is similarly controlled by the −16kb enhancer. Thus, this novel enhancer‐deleted murine model may be useful in future studies for examining the role of FGF23 elevations in CKD and other inflammatory diseases.

## Disclosures

All authors state that they have no conflicts of interest.

## Supporting information

Supporting Figure S1.

Supporting Figure S2.

Supporting Figure S3.

Supporting Figure S4.

Supporting Table S1.

Supporting Table S2.
