# Supplementary material for: A Novel Distal Enhancer Mediates Inflammation‐, PTH‐, and Early Onset Murine Kidney Disease‐Induced Expression of the Mouse Fgf23 Gene
Source: JBMR Plus. 2017 Nov 21;2(1):31–46. doi: 10.1002/jbm4.10023 (PMC5842943; doi:10.1002/jbm4.10023)
Supplement: Supplementary file 5 — Supporting Table S1. [file JBM4-2-31-s005.docx]

**Supplementary Table 1 . Matrix families used in de novo analysis**

| **Nuclear factor kappa B/c-rel** | |  | **Hypoxia inducible factor,**  **bHLH/PAS protein family** | |
| --- | --- | --- | --- | --- |
| **Matrix name** | **IUPAC** |  | **Matrix name** | **IUPAC** |
| V$CREL.01 | BGGGNTTTCC |  | V$ARNT.01 | NDDNNCACGTGNN |
| V$HIVEP1.01 | WDGGGAMTTTCCN |  | V$CLOCK_BMAL1.01 | GGGTCACGTGNN |
| V$NFKAPPAB.01 | GGGANTYYCC |  | V$HIF1.01 | HSBCGBACGTGNS |
| V$NFKAPPAB.02 | NGGGACTTTCCN |  | V$HIF1.02 | BVCHBACGTGS |
| V$NFKAPPAB50.01 | GGGGATYCCC |  | V$HRE.01 | NGKNNNKACGTGCGNN |
| V$NFKAPPAB65.01 | BGGRRTTTCC |  | V$HRE.02 | NNACGTGNN |
| V$NFKAPPAB65.02 | NNGGGGATTTCCCNN |  |  |  |

| **Signal transducer and activator of transcription** | |
| --- | --- |
| **Matrix name** | **IUPAC** |
| V$STAT.01 | TTMYGGGAA |
| V$STAT1.01 | ANTTCCGGGAANTGNS |
| V$STAT1.02 | NNTTCCAGGAANN |
| V$STAT3.01 | NNATTTCCSGGAARTGNN |
| V$STAT3.02 | NNTTCCWGGWMNN |
| V$STAT5.01 | NNTTCYNRGAARWNN |
| V$STAT6.01 | RNYTTCCYRRGAANN |
