# Supplementary material for: A Novel Distal Enhancer Mediates Inflammation‐, PTH‐, and Early Onset Murine Kidney Disease‐Induced Expression of the Mouse Fgf23 Gene
Source: JBMR Plus. 2017 Nov 21;2(1):31–46. doi: 10.1002/jbm4.10023 (PMC5842943; doi:10.1002/jbm4.10023)
Supplement: Supplementary file 6 — Supporting Table S2. [file JBM4-2-31-s006.docx]

| ***Fgf6*** | **Bone (x10^4^)** | | **Thymus (x10^4^)** | | **Spleen (x10^5^)** | | **Kidney** | | **Intestine** | | **Liver** | |
| --- | --- | --- | --- | --- | --- | --- | --- | --- | --- | --- | --- | --- |
|  | **veh** | **LPS** | **veh** | **LPS** | **veh** | **LPS** | **veh** | **LPS** | **veh** | **LPS** | **veh** | **LPS** |
| **WT** | 7.9+1.1 | 2.8+1.2* | 4.0+1.3 | 19.1+6.8* | 0.7+0.1 | 3.2+1.2 | U.D. | U.D. | U.D. | U.D. | U.D. | U.D. |
| ***Fgf23*^-16ko^** | 5.1+1.4^#^ | 2.2+0.3* | 3.0+1.1 | 17.5+3.9* | 0.8+0.4 | 4.0+2.1 | U.D. | U.D. | U.D. | U.D. | U.D. | U.D. |
|  |  |  |  |  |  |  |  |  |  |  |  |  |
|  | **veh** | **IL-1β** | **veh** | **IL-1β** | **veh** | **IL-1β** | **veh** | **IL-1β** | **veh** | **IL-1β** | **veh** | **IL-1β** |
| **WT** | 3.5+3.3 | 4.2+6.7 | 6.4+2.2 | 5.6+1.2 | 2.1+1.1 | 1.1+1.2 | U.D. | U.D. | U.D. | U.D. | U.D. | U.D. |
| ***Fgf23*^-16ko^** | 3.6+8.0 | 7.9+10.2 | 3.7+2.1 | 4.0+1.1 | 2.0+1.5 | 0.6+0.5 | U.D. | U.D. | U.D. | U.D. | U.D. | U.D. |
|  |  |  |  |  |  |  |  |  |  |  |  |  |
|  | **veh** | **Tnfα** | **veh** | **Tnfα** | **veh** | **Tnfα** | **veh** | **Tnfα** | **veh** | **Tnfα** | **veh** | **Tnfα** |
| **WT** | 7.9+2.3 | 6.4+2.1 | 3.3+0.8 | 2.1+1.5 | 0.1+0.2 | 0.3+0.4 | U.D. | U.D. | U.D. | U.D. | U.D. | U.D. |
| ***Fgf23*^-16ko^** | 6.8+1.8 | 5.9+1.5 | 2.1+1.1 | 2.2+1.4 | 0.5+0.6 | 0.1+0.2 | U.D. | U.D. | U.D. | U.D. | U.D. | U.D. |
|  |  |  |  |  |  |  |  |  |  |  |  |  |
| ***Tigar*** | **Bone (x10^4^)** | | **Thymus (x10^4^)** | | **Spleen (x10^4^)** | | **Kidney (x10^3^)** | | **Intestine (x10^4^)** | | **Liver (x10^3^)** | |
|  | **veh** | **LPS** | **veh** | **LPS** | **veh** | **LPS** | **veh** | **LPS** | **veh** | **LPS** | **veh** | **LPS** |
| **WT** | 8.4+1.0 | 8.1+1.2 | 2.5+0.2 | 2.8+0.1 | 4.1+0.2 | 3.9+0.3 | 1.6+0.1 | 0.8+0.0* | 6.3+0.3 | 4.3+0.1* | 4.2+0.6 | 1.5+0.3* |
| ***Fgf23*^-16ko^** | 7.3+1.5 | 8.2+0.3 | 2.5+0.2 | 2.7+0.2 | 3.9+0.3 | 3.6+0.6 | 1.8+0.1 | 0.8+0.1* | 6.0+0.4 | 4.0+0.6* | 4.1+0.6 | 1.7+1.0* |
|  |  |  |  |  |  |  |  |  |  |  |  |  |
|  | **veh** | **IL-1β** | **veh** | **IL-1β** | **veh** | **IL-1β** | **veh** | **IL-1β** | **veh** | **IL-1β** | **veh** | **IL-1β** |
| **WT** | 9.4+0.8 | 6.2+0.4 | 2.6+0.5 | 3.2+0.5 | 3.3+0.3 | 3.6+0.4 | 1.8+0.2 | 1.4+0.1* | 4.7+0.3 | 3.3+0.3* | 3.2+0.6 | 2.6+0.5 |
| ***Fgf23*^-16ko^** | 9.0+0.8 | 7.4+1.3 | 2.9+0.4 | 2.8+0.4 | 3.2+0.4 | 3.8+0.1 | 1.8+0.2 | 1.5+0.2 | 4.6+0.5 | 3.2+0.2* | 3.0+0.5 | 2.7+0.7 |
|  |  |  |  |  |  |  |  |  |  |  |  |  |
|  | **veh** | **Tnfα** | **veh** | **Tnfα** | **veh** | **Tnfα** | **veh** | **Tnfα** | **veh** | **Tnfα** | **veh** | **Tnfα** |
| **WT** | 3.0+0.7 | 3.3+0.6 | 1.2+0.2 | 1.1+0.1 | 1.6+0.1 | 1.6+0.2 | 1.8+0.1 | 1.1+0.1* | 5.7+0.5 | 4.7+0.4* | 2.8+0.5 | 1.9+0.2* |
| ***Fgf23*^-16ko^** | 2.6+0.6 | 2.9+0.5 | 1.2+0.3 | 1.1+0.2 | 1.5+0.2 | 1.4+0.1 | 1.7+0.1 | 1.2+0.1* | 5.6+0.4 | 4.4+0.5* | 2.4+0.5 | 1.7+0.3* |

**Supplementary Table 2. Lack of -16kb enhancer does not mediate expression of *Fgf6* or *Tigar***
